# Genome-wide search for breast cancer linkage in large Icelandic non-BRCA1/2 families

**DOI:** 10.1186/bcr2608

**Published:** 2010-07-16

**Authors:** Adalgeir Arason, Haukur Gunnarsson, Gudrun Johannesdottir, Kristjan Jonasson, Pär-Ola Bendahl, Elizabeth M Gillanders, Bjarni A Agnarsson, Göran Jönsson, Katri Pylkäs, Aki Mustonen, Tuomas Heikkinen, Kristiina Aittomäki, Carl Blomqvist, Beatrice Melin, Oskar TH Johannsson, Pål Møller, Robert Winqvist, Heli Nevanlinna, Åke Borg, Rosa B Barkardottir

**Affiliations:** 1 Department of Pathology, Landspitali-LSH v/Hringbraut, 101 Reykjavik, Iceland; 2 Faculty of Medicine, University of Iceland, Vatnsmyrarvegi 16, 101 Reykjavik, Iceland; 3 Faculty of Engineering and Natural Sciences, University of Iceland, Hjardarhaga 2-4, 107 Reykjavik, Iceland; 4 Department of Oncology, Clinical Sciences Lund, Lund University, SE 221 85 Lund, Sweden; 5 Inherited Disease Research Branch, National Human Genome Research Institute, National Institutes of Health, 333 Cassell Drive, Suite 1200, Baltimore, MD 21224, USA; 6 Laboratory of Cancer Genetics, Department of Clinical Genetics and Biocenter Oulu, University of Oulu, Oulu University Hospital, 90220 Oulu, Finland; 7 Department of Obstetrics and Gynecology, Helsinki University Central Hospital, P.O. BOX 700, 00029 HUS, Helsinki, Finland; 8 Department of Clinical Genetics, Helsinki University Central Hospital, P.O. BOX 140, 00029 HUS, Helsinki, Finland; 9 Department of Oncology, Helsinki University Central Hospital, P.O. BOX 180, 00029 HUS, Helsinki, Finland; 10 Department of Radiation Sciences, Umeå University, 901 85 Umeå, Sweden; 11 Department of Oncology, 20A, Landspitali-LSH v/Hringbraut, 101 Reykjavik, Iceland; 12 Section of Inherited Cancer, Oslo University Hospital, 0310 Oslo, Norway

## Abstract

**Introduction:**

A significant proportion of high-risk breast cancer families are not explained by mutations in known genes. Recent genome-wide searches (GWS) have not revealed any single major locus reminiscent of *BRCA1 *and *BRCA2*, indicating that still unidentified genes may explain relatively few families each or interact in a way obscure to linkage analyses. This has drawn attention to possible benefits of studying populations where genetic heterogeneity might be reduced. We thus performed a GWS for linkage on nine Icelandic multiple-case non-*BRCA1/2 *families of desirable size for mapping highly penetrant loci. To follow up suggestive loci, an additional 13 families from other Nordic countries were genotyped for selected markers.

**Methods:**

GWS was performed using 811 microsatellite markers providing about five centiMorgan (cM) resolution. Multipoint logarithm of odds (LOD) scores were calculated using parametric and nonparametric methods. For selected markers and cases, tumour tissue was compared to normal tissue to look for allelic loss indicative of a tumour suppressor gene.

**Results:**

The three highest signals were located at chromosomes 6q, 2p and 14q. One family contributed suggestive LOD scores (LOD 2.63 to 3.03, dominant model) at all these regions, without consistent evidence of a tumour suppressor gene. Haplotypes in nine affected family members mapped the loci to 2p23.2 to p21, 6q14.2 to q23.2 and 14q21.3 to q24.3. No evidence of a highly penetrant locus was found among the remaining families. The heterogeneity LOD (HLOD) at the 6q, 2p and 14q loci in all families was 3.27, 1.66 and 1.24, respectively. The subset of 13 Nordic families showed supportive HLODs at chromosome 6q (ranging from 0.34 to 1.37 by country subset). The 2p and 14q loci overlap with regions indicated by large families in previous GWS studies of breast cancer.

**Conclusions:**

Chromosomes 2p, 6q and 14q are candidate sites for genes contributing together to high breast cancer risk. A polygenic model is supported, suggesting the joint effect of genes in contributing to breast cancer risk to be rather common in non-*BRCA1/2 *families. For genetic counselling it would seem important to resolve the mode of genetic interaction.

## Introduction

Increased susceptibility to breast cancer (BC) has been shown to be caused by germline segregation of three different classes of alleles: 1) high-penetrance genes with rare risk variants, 2) moderate-penetrance genes, also with rare variants and 3) low-penetrance alleles of common frequency [1]. Hereditary BC, defined by a significant familial aggregation of BC and explaining approximately 5 to 10% of cases diagnosed with BC, is as yet seen to arise from the first allele class whenever the causative gene is known. Genetic counselling can then be provided, based on mutation screening. A significant proportion of the families are not associated with mutations in *BRCA1 *or *BRCA2 *or other known genes [2-5] and may in part be explained by recessive alleles or a polygenic model with risk variants of lower penetrance jointly affecting risk in miscellaneous combinations [6-8]. However, the gene most recently identified, *RAD51C *[9], demonstrates that some proportion may still have a high-risk-allele cause. *RAD51C *was identified using a candidate gene approach, but if more high-penetrance genes are yet to be identified it might also be helpful to analyse families from populations where genetic heterogeneity might be reduced [10,11]. Recent genome-wide searches (GWS) for BC linkage in families without alterations in known genes (non-*BRCA1/2 *families) [10-16], together with earlier suggestions of single loci [17-19], indicate close to 20 candidate chromosome regions if accepting LOD scores ≥ 1.5 found in a single family or small group of families [16]. Two regions have been independently pinpointed by more than one study, one on chromosome 2p21 to p22 [11,13] and the other at 6q24 where the *ESR1 *gene is located [16,19]. In some studies, notable linkage signals have been seen at two or three chromosome regions in the same family [11-13,16], which by chance would have a low probability [15].

Two issues helped shape our current GWS study. First, both *BRCA1 *and *BRCA2 *are tumour suppressor genes and most often involve wild-type loss of heterozygosity (wt-LOH) in mutation carriers' tumours [20,21], resulting in both parental copies of the gene being damaged in line with Knudson's two-hit model [22]. Any new loci suggestive by large families to confer high-risk of BC would predictably gain support from the observation of such a wt-LOH signature; on the other hand, the lack of it would leave open the question of different gene functions. Second, in Iceland both *BRCA1 *and *BRCA2 *have been found with recurrent mutations, one in each gene, with the *BRCA2-999del5 *mutation occurring in 8.5% of BC patients and 0.5% of the population [23-25]. Other mutations in these genes in Iceland have not been published and would presumably be very rare [3]. This accords with the relative geographical isolation of the Icelandic population which since the settlement of the island in the ninth century has at times suffered famine- or epidemic-caused reductions (with population size only 38,000 in the year 1800 compared to the current size of 319,000), which in effect should reduce the complexity of a gene search. We therefore selected nine large Icelandic non-*BRCA1/2 *families for a GWS of high-risk genes under a parametric dominant linkage model. Regions considered suggestive (LOD ≥ 1.5 per family) were also subjected to wt-LOH analyses in tumours from putative gene carriers, and the same regions were genotyped in a collection of Nordic non-*BRCA1/2 *families, in order to estimate the possible proportion of linked families in Iceland and other Nordic countries.

## Materials and methods

After screening for recurrent Icelandic *BRCA1 *and *BRCA2 *mutations in 438 BC cases diagnosed in Iceland in the years 1989 to 2001, the history of BC was evaluated in the pedigrees of both the mother's and the father's family side of the non-*BRCA1/2 *cases. Nine families were selected and subjected to a GWS of BC linkage by the selection criteria of (1) at least three women diagnosed with BC under age 60 years (omitting bilineal cases), (2) the availability of blood or paraffin-embedded normal tissue for isolation of DNA of sufficient quality from at least four affected cases (any age), and (3) evidence against linkage to *BRCA1 *or *BRCA2 *according to genotyped microsatellite markers flanking and within these genes. Each of the nine families consisted of descendents of a single pair of founders. In five families a DNA sample was available from six or more BC cases (Additional file 1, Figure S1). In the analyses, the nine families were treated as 12 because three pedigrees (70070, 70228 and 70236) were too large for the GWS linkage analysis software and were therefore separated by branches in two parts each (Additional file 1, Figure S1). The two family sides were compared by inspection of LOD signals (selection of peaks based on NP-LOD related *P*-values) in order to find possibly overlapping positions, which could then be further examined by manual comparison of haplotypes.

Thirteen additional Nordic families were used in follow-up studies on suggestive loci on chromosomes 2p, 6q and 14q. They were selected from available non-*BRCA1/2 *families at other Nordic centres, in line with the above criteria and in such a way that the genotyped affected family members would not be expected to share by descent more than approximately 6% of alleles by chance (through at least six meioses). Written informed consent was obtained with all blood samples and appropriate Institutional Review Board approvals were obtained. Characteristics of the 22 families are summarised in Table 1 and further details about the families included in the GWS are provided in Figure S1 (Additional file 1).

**Table 1:** Summary of families by group

	Number of families						
		
Group	Total	Number of cases of BC			Cases with age at onset < 50 y		Number of genotyped individuals (affected)
				
		4	5	6+	< 4	4+	
Iceland*	9 (12)	0 (1)	2 (6)	7 (5)	6 (10)	3 (2)	102 (60)
Lund/Oslo	3			3	1	2	31 (14)
Helsinki	7	1	4	2	5	2	56 (31)
Oulu	3	1	1	1	3		20 (15)
Total	22	2	7	13	15	7	208 (119)

* Except for the last column, numbers within parentheses refer to the number of families as processed in linkage calculations, in which three families were separated in two parts each due to large size (see Additional file 1, Figure S1).

DNA was extracted from nuclei of lysed blood samples according to Miller *et al*. [26] or by standard phenol-chloroform extraction, from fresh-frozen tissue using the Wizard Genomic DNA Purification Kit (Promega, Madison, Wisconsin, USA) and from paraffin-embedded tissue using a xylene treatment followed by proteinase K digestion and phenol/chloroform/isoamyl alcohol purification. All genotyping was performed at the same centre; each sample plate contained a blank well, two duplicate samples and a Centre d'Etude du Polymorphisme Humain (CEPH) control. Samples included in the GWS were genotyped using the Applied Biosystems (Foster City, California, USA) HD-5 Linkage Mapping Set, containing 811 fluorescently labelled PCR primer pairs that define an approximately five centiMorgan (cM) resolution human index map. Genotypes were analysed using an automated ABI PRISM 3130 × l Genetic Analyzer with GeneMapper software v4.0 (Applied Biosystems, Foster City, California, USA) for automatic calling of alleles, and then checked manually. For LOH analysis (eight members of family 70234), DNA was also isolated from tumour tissue, which was obtained from paraffin blocks (invasive primary tumours) after selecting areas rich in tumour cells (> 90%) by microscopy (all by the same investigator) and relative allele intensities were then compared to those of blood or normal-tissue from the same individual. For six women, this tumour DNA or DNA from fresh-frozen tissue was also subjected to array comparative genomic hybridisation (array-CGH). Arrays were produced at SCIBLU Genomics, Lund University as previously described [27] using the 32K tiling BAC clone set from the CHORI BACPAC resource centre.

Merlin software (Center for Statistical Genetics, University of Michigan, Ann Arbor, Michigan, USA) [28] was used for the linkage analysis. Four different multipoint analyses were carried out and associated LOD scores calculated: (i) parametric dominant and (ii) recessive with age dependent liability classes (14 total) as defined using the modified Cancer and Steroid Hormone Study (CASH) model [29]; cf. [4,30], (iii) non-parametric using S-all scoring [31] and (iv) S-pairs scoring [32], in both cases with the exponential model [33]. Only cases with invasive BC were coded as affected; all other cancers were assigned with unknown status. Two affected cases in one family (70234) were identical twins and only one of them was included in linkage calculations. For parametric linkage heterogeneity LOD scores (HLOD) are reported. Under the parametric models disease allele frequencies of 0.0033 (dominant) and 0.08 (recessive) were assumed as in [29]. The Rutgers Map v.2 [34] (which is based on the deCODE map [35]) was used to locate markers, and if not present in that map they were placed with linear interpolation using their physical position in base pairs relative to flanking markers. Allele frequencies were estimated separately for each country by counting in all individuals (the -fa option of Merlin). LOD scores for individual families were also calculated, by running each family separately in Merlin, but using allele frequencies of the total sample of the relevant country. Genotypes that were incompatible with the family relations (inheritance errors), as well as unlikely genotypes, were eliminated with the help of Merlin software.

In order to analyse conditional probabilities by family of being *linked *under the admixture model, the files prepared by Merlin software were reformatted to fit LINKMAP software (National Center for Biotechnology Information (NCBI), Bethesda, Maryland, USA) for sliding three-point linkage analysis of selected markers. Eighteen markers were analysed at 6q, 16 at 14q and 7 at 2p, using country-specific allele frequencies. Disease allele frequency and age dependent liability classes were as above, under the dominant model [29,30].

The probability of two or three haplotypes from independently segregated chromosomal regions (*a priori *unknown positions), being simultaneously cosegregated with a trait through *m *meioses, in one out of *N *families with similar or greater total number of meioses (Bonferroni adjustment), was calculated as described (Additional file 2). Briefly, the resulting *P*-value for two loci is(1)

and for three loci(2)

## Results

**Figure 1:**
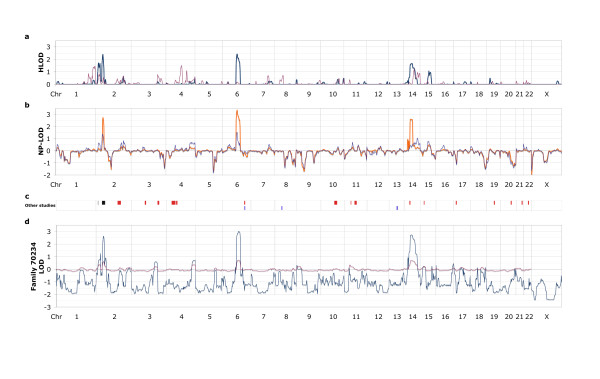
**Maximum LOD scores by chromosomal position, and relation to previously suggested candidate loci**. Parametric HLOD scores for the nine Icelandic families are shown in **(a) **for the dominant (dark teal thick line) and the recessive model (plum). NP-LOD scores are shown in **(b) **using different exponential scoring options in Merlin software: S-all (orange thick line) and S-pairs (indigo). The position of previously published loci is shown in **(c)**, according to GWS studies in red (or black if reported in more than one GWS study) (adapted from Table 5 in [16]), and according to single locus reports [17-19] in blue. A position at 2p indicated by a subset of relatively early-onset multiple-case families in one GWS-study [11] is included in (c) and shown in grey. Parametric LOD scores of family 70234 are shown in **(d) **with line colours as in (b).

Nine Icelandic BC families, unexplained by *BRCA1 *or *BRCA2 *mutations (non-*BRCA1/2 *families), were genotyped for a set of 811 genome-wide distributed microsatellite markers for subsequent linkage analysis. As all pedigrees appeared high-risk and dominant, the main analysis was based on the parametric dominant model but other models were also considered in order to see if the LOD scores were sensitive to model misspecification. Figure 1a, b shows LOD scores for parametric and non-parametric linkage analysis by chromosomal position for the nine families combined. For comparison, the position of the main indications of BC susceptibility according to previously published studies of non-*BRCA1/2 *linkage is shown in Figure 1c. Two of three regions with dominant HLOD > 1.5 overlap with previously indicated loci. One of them maps to 2p and contains two separate peaks (peak HLOD 1.72 at D2S162 and 2.41 at D2S367; both peaks overlapping with previous indications) and the other to 14q (HLOD 1.68 at D14S63). However, the third region, at 6q (reaching HLOD 2.44 at D6S300) does not overlap with previously reported candidates (Figure 1a-c).

**Figure 2:**
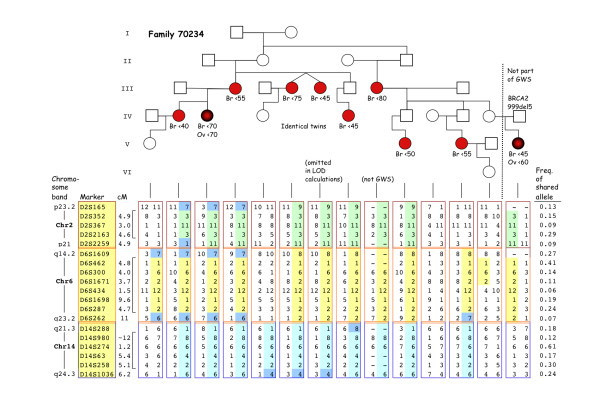
**Cosegregation of haplotypes at three chromosomal regions in family 70234**. On top, the pedigree of this family is shown with circles denoting females and boxes males, with red filling denoting diagnosis of BC and shaded red also ovarian cancer. The pedigree is somewhat distorted in order to avoid recognition, but preserves the number of male and female meioses. Information about approximate age (in years) at diagnosis of cancer is shown below symbols (Br for breast and Ov for ovarian). One woman inherited a 999del5 BRCA2 mutation from her father (red box), not otherwise blood-related to this family. This woman and one other (denoted by *not GWS*) were not included in the genome-wide search. One of a pair of identical twins was omitted in linkage calculations. Under the pedigree, genotypes of markers of interest are shown (with chromosomal band position, marker names and genomic distance shown at the left, brackets indicating position between sites of relevant recombination). Colouring of alleles denotes whether they belong to a shared haplotype (allele frequency shown in the rightmost column) or derive from a recombined chromosome (blue). Plain (not coloured) alleles denoted 11 and 3 at D2S367 and D2S2163 respectively, are identical by state, but probably not by descent, to the commonly segregated allele (conclusion supported by fine-genotyping of additional markers, data not shown). The low frequency (0.06) of the shared D6S434 allele was validated by typing in 59 unrelated Icelandic control individuals. This figure was 0.02 in the controls (2/118), which coincides with the published Genethon frequency of the allele.

When analysing separate families, one (named 70234) was seen to contribute high scores (LOD 2.63 to 3.03) at all three major positions, 2p, 6q and 14q (Figure 1d). At 6q and 14q haplotypes of 29 and 13 Mb DNA, respectively, were shared by all nine non-*BRCA1/2 *BC-affected women in this family and eight of these women also shared a haplotype (9 Mb) at 2p (Figure 2). With reference to the flanking (recombined) markers, the common haplotypes map to 2p23.2 to p21 (between D2S165 and D2S2259), 6q14.2 to q23.2 (between D6S1609 and D6S262) and 14q21.3 to 24.3 (between D14S288 and D14S1036). A close inspection of the information in Figure 2 makes it is evident that the 6q and 14q haplotypes are identical by descent and have cosegregated through 13 meioses, and that the 2p haplotype has cosegregated with the other two through 11 meioses. Of the nine families, a total of five had a comparable total number of meioses connecting the affected cases (ranging from 13 to 18). Therefore a Bonferroni correction of *N *= 5 has been chosen. By formula (1) the probability of observing cosegregation of two loci (6q and 14q) through 13 meioses in one of five families is *P *= 0.006. The corresponding probability for all three loci and 11 meioses is also *P *= 0.006 (formula (2)). We also used Merlin to simulate marker data in order to get an indication of the frequency of obtaining three separate dominant model LOD scores > 2.5. A total of 700 replicates of the per-family runs were generated using the same allele frequency estimates as in the original run. In none of the replicates did we obtain a family with three peaks of this magnitude, in two instances there was a family with two peaks, in 75 instances we obtained a family with one location with dominant LOD > 2.5 (in six instances a family exceeding LOD 3.0), and in the remaining simulated cases no dominant LOD > 2.5 was obtained.

Eight breast tumours from family 70234 were assayed at markers within the shared haplotypes, for loss of heterogeneity selective for losing wild-type alleles (wt-LOH) in harmony with Knudson's model of tumour suppressor genes. At 6q, LOH was seen at all informative markers in three tumours, with wt-LOH in two but the third lost all alleles from the risk-related haplotype (data not shown). Copy-number loss of the region was confirmed in the three tumours by array-CGH (data not shown) which also revealed amplification at 6q21 in a fourth tumour. At 2p and 14q, signs of allelic losses were confined to single markers and of inconsistent allelic phase (three tumours each chromosome), and not supported by array-CGH since intensities were generally within thresholds.

Family-wise, no other family than 70234 showed parametric LOD scores higher than 1.5 (and the highest NP-LOD for the other families was 2.3). LOD scores of weaker indication were seen at multiple positions (Additional files 3 and 4, Figures S2 and S3). In order to see if any position might be indicated by more than one family, even if too weakly suggestive on a single-family basis, we listed all peaks that met the criterion of NP-LOD associated *P*-values of < 0.005 (Additional file 5, Table S1). Of 22 peaks, one was found to colocate with that of family 70234 to the 6q15 to q22 region and two families shared peaks at 13q32.1 to q33.1. In case of separate parts of the same pedigree, possibly overlapping peaks were not observed.

To follow up suggestive linkage signals (HLOD > 1.5 by family), markers at 2p, 6q and 14q were genotyped in an additional 13 Nordic non-*BRCA*1/2 families. Dominant parametric HLOD scores for the 13 families combined reached 0.92 at D6S434, and were much lower at 2p and 14q (highest HLOD 0.23 at D2S165). An analysis of heterogeneity, using LINKMAP was performed on all 22 Nordic families (treated as 25 for the reasons given above) at the three chromosomal positions and the results are shown in Table 2. At chromosome 6q15 to q22.31, the combined HLOD was 3.27 and α 0.40. The data show support for chromosome 6q-linkage among both Icelandic and non-Icelandic families. HLODs by country subset were in the range 0.34 to 2.02 (α ranging between 0.39 and 1). Single families with a conditional probability of being linked exceeding the overall α-value of 0.40 (10 families) were widely distributed among the Nordic subsets (Table 2). At chromosome 14q21.2 to q24.3 the main support for linkage lies within the Icelandic subset (Table 2) with HLOD 1.24 and α 0.36, unaltered by the addition of other Nordic families. At 2p23.2 to p21 most non-Icelandic families fail to support the findings since they indicate a more telomeric position (Table 2) which does not overlap with the high peak in family 70234. An exception is one Finnish family with LOD 1.03, matching the position in family 70234. It has a conditional probability of being linked equal to 0.78 (data not shown), only exceeded by that of family 70234 (0.97).

**Table 2:** Peak parametric multipoint LOD scores under heterogeneity, at three chromosomal regions as defined by family-70234 haplotypes

		6q15 to q22.31				14q21.2 to q24.3				2p23.2 to p21			
				
Subset	Number of families*	HLOD	α	**cM from D6S434**^**†**^	**Number of linked families**^**‡**^	HLOD	α	**cM from D14S980**^**†**^	**Number of linked families**^**‡**^	HLOD	α	**cM from D2S367**^**†**^	**Number of linked families**^**‡**^
Icelandic	12	2.02	0.45	-5.4	3	1.24	0.36	30.2	4	2.13	0.61	0.0	7
Lund/Oslo	3	1.37	0.79	0.3	2	0			0	0.46	1.00	-14.4	0
Helsinki	7	0.34	0.39	12.2	3	0.06	0.34	22.2	0	1.09	1.00	-13.1	2
Oulu	3	1.15	1.00	-1.9	2	0			0	0.75	1.00	-7.5	1
Total	25	3.27	0.40	0.0	10	1.24	0.36	30.2	4	1.66	0.25	0.0	10

*Three Icelandic pedigrees were separated in two parts each in the linkage calculations. Therefore the total number of families in this table is 25.

^†^For each locus, the distance relates to the marker closest to the LOD-peak in family 70234; negative numbers refer to the direction towards the p-telomere.

^‡^Linked families are defined as having a conditional probability of linked type (data not shown) exceeding the alpha for all families combined.

## Discussion

In the present GWS for BC linkage in nine Icelandic non-*BRCA1/2 *families, substantial linkage signals were observed at chromosomes 2p25.1 to p22.1, 6q15 to q22.31 and 14q21.3 to q31.3, and the strongest contribution to all three regions occurred in the same family (70234). On a single family basis, all three signals in the family are exceptionally high compared to previous studies [10-19], but none of the signals meet the suggested cut off level of 3.3 for significance in GWS studies [36]. The regions at 2p and 14q overlap with those of previous studies of non-*BRCA1/2 *families. The position of the more centromeric signal in our 2p region (2p23.3 to p21) was in fact pinpointed on the basis of single families by two independent studies [11,13]. The more telomeric signal in our 2p region (p25.1 to p24.1) contains a position with relation to families with a higher number of cases and at a younger age at diagnosis [11]. The region at 6q15 to q22.31 does not extend to the 6q24.3 to q25.1/ESR1-region [16,19] and does therefore not overlap with previous indications. This region had the strongest signal (HLOD 3.27 in all families combined) in the current study and gains support from some other families besides 70234, both Icelandic and from other Nordic countries (Table 2).

Recent studies have indicated familial non-*BRCA1/2 *BC as mainly polygenic with decreasing possibility of finding new high-risk genes. Our results support this view in the following way: The current study was primarily designed to find whether dominant mutations of high penetrance exist in large Icelandic non-*BRCA1/2 *families with a Mendelian pattern concurrent with such genetic explanation. Most families failed to reveal evidence of any such locus. Although three chromosomes provided suggestive linkage signals, their coexistence in one family hardly supports the idea of a single causative gene. Some families have previously been reported with two or three suggestive chromosome regions [11-13,16] and such an observation was shown to have a low probability simply by chance [15]. We do not consider chromosomal translocation to be a logical possibility since the three haplotypes in question segregate independently to the daughters of affected cases in family 70234 (Figure 2). We estimate the probability to be *P *= 0.006 of seeing any two loci that are not linked to each other cosegregate by chance with the disease through 13 meioses as here seen at 6q and 14q. This would argue for the existence of more than one causative gene in family 70234. Whether the third locus (2p), which also cosegregates to eight of the nine cases, plays a role in the risk is a little more ambiguous. Although the ninth case inherited some alleles identical with the common haplotype (and therefore contributed to the high LOD of 2.63) they were on closer scrutiny (genotyping of added markers, data not shown) seen to be interrupted by non-matching alleles and therefore she should be regarded as a phenocopy with respect to possible 2p-linkage. By formula (2), the probability of observing cosegregation of three loci through 11 meioses in one of five families is *P *= 0.006. Although this might be adjusted with respect to different possible ways of observing a phenocopy, and to the fact that the phenocopy did inherit two of the three haplotypes, the resulting value would still be on the same order as the compared value for two loci. The 2p-haplotype may therefore be irrelevant to the BC risk but it gains support from the two previous reports of a candidate BC susceptibility locus which overlaps with this 2p-region [11,13].

It may be asked if the unique triple strong signals in family 70234 as compared to other families possibly reflect distinction by genetic linkage models. The absence of notable linkage peaks in the remaining families conforms to a polygenic model with frequent alleles, sometimes cosegregating and sometimes being replaced by variants of different lineage or of other genes. Family 70234 seems to differ in this respect, even if questioning the 2p-linkage, since the two haplotypes at 6q and 14q fully cosegregate with the disease in nine cases, with maximum LOD ranging from 2.74 to 3.03. Therefore a replacement of one haplotype by a new variant (if needed) would appear to be a rare event in this instance, and one might expect a low population frequency of the gene variants involved. This would have implications for genetic counselling, since continued cosegregation of the two (not to mention three) haplotypes would seem improbable for the descendants of family 70234. It would then also be important to resolve whether each of the genetic variants contributes an independent proportion of the disease risk (additive or multiplicative joint effects), or in an interdependent way, their risk being dependent on the presence of all cofactors. With such a scenario, one would view this family's cancer history mainly as a very rare chance result of cosegregation of limited consequence for later generations of the family. Alternatively, the families studied here may all comply with the same genetic model, with cooperative alleles of moderate or high frequency, and the strong linkage signals in family 70234 to be accounted for by the absence of phenocopies. We note, in this context, that by treating the nine families as 12 for linkage calculations, most were not as highly informative by the number of cases or meioses as 70234, but nevertheless they were expected to reveal clues of genomic position by pairwise comparison of separate parts of the larger pedigree.

The additional Nordic families support the risk indication of chromosome 6q, but seem not to support the 2p- and 14q-linkage. One Finnish family may be of the linked type at the same 2p position as family 70234 but this is not higher than expected by chance from 13 families, each with up to 6% sharing, by descent, of genetic material between its affected members. The 6q-linkage gains some support from other Icelandic families (for example, 70386 in Additional file 5, Table S1). This raises the question whether a recurrent mutation may be involved, but comparison of haplotypes in suggestive carriers from different families did not support that idea (data not shown). If present, such a recurrent mutation would seemingly call upon the genotyping of more densely distributed markers.

At chromosome 2p, a linkage signal in the combined families, telomeric to the one in family 70234 and concurrent with the reported position of a signal in relatively early-onset multiple-case families [11], also invites searching for an underlying recurrent mutation in the Icelandic families. However, although some families contribute to this signal with weakly positive LOD scores (Additional files 3 and 4, Figures S2, S3), they lack reliable indications of which alleles to look for, partly due to absence of a convincing 'reference' family (like 70234 in the case of 6q) and partly due to uncertain recombination events, and such comparison of haplotypes is therefore meaningless. In short, a sign of a recurrent mutation (that is, alleles of not too high frequency, seen in different candidate families) would support a risk related role of this locus, but it is not seen.

As regards the three chromosome regions most strongly indicated in our study, we tested by wt-LOH analysis whether the genes in question might act as classical tumour suppressors. Three out of eight tumours from family 70234 showed extensive LOH at 6q but it affected both wild-type and risk related haplotypes. At 2p and 14q, convincing signs of LOH were absent. Therefore no support was found for the hypothesis of a predisposing tumour suppressor gene similar to the *BRCA1 *and *BRCA2 *genes [20,21]. We note, however, that the hypothesis is not ruled out because microalterations could exist that are not seen by our methods.

With respect to the four different ways used to analyse linkage, we had reasons to choose the parametric dominant analyses as the principal one. Looking at the pedigrees none appeared recessive. We also expected low genetic heterogeneity. That would argue for s-all to be the primary non-parametric method but for comparison we also performed s-pairs analyses. By considering all four analyses (Figures 1 and Additional files 3 and 4, Figures S2, S3), the parametric dominant analyses did not appear to be sensitive to model misspecification.

The results of our GWS analysis support previously reported indications of a polygenic nature of non-*BRCA1/2 *hereditary BC. Most families in the current study fail to provide map indications of involved loci and this may in part be credited to the problem of phenocopies, which was addressed by simulation experiments on *BRCA1/2 *families in the GWS study of Rosa-Rosa *et al. *[16]. Finding more families with signals analogous to those of 70234 in the current study could provide further clues where to look for interacting risk loci and a follow-up of more generations could then help to resolve the significance and mode of possible genetic interaction.

## Conclusions

The results of this study support previous indications that susceptibility to BC in multiple-case non-*BRCA1/2 *families seems to be segregated by low- or moderate-penetrance gene variants jointly contributing to the risk. A combination of variants at chromosomes 2p, 6q and 14q may in a cooperative or even interdependent way cause high disease risk in a family. Together with other such families reported with multiple linkage signals, this may reflect localised familial clustering of risk alleles from a pool of many candidate loci. Genetic counselling would benefit from resolving the mode of interactions in such families.

## Abbreviations

BC: breast cancer; GWS: genome-wide search/genome-wide scan; HLOD: heterogeneity LOD (parametric); LOD: logarithm of odds; LOH: loss of heterozygosity; non-*BRCA1/2*: not accounted for by mutations in *BRCA1 *or *BRCA2 *or other known genes; NP-LOD: non-parametric LOD; wt-LOH: LOH with loss from the "wild-type" chromosome.

## Competing interests

The authors declare that they have no competing interests.

## Authors' contributions

RBB and AA designed the study and together with EG selected the Icelandic families after an initial power simulation performed by EG. HG and GJo carried out the DNA analysis and genotype data collection and AA did the processing and checking of the data. KJ carried out the linkage analyses and significance testing, helped with organising and writing statistical parts of the manuscript, and together with AA, shaped the statistical analysis of cosegregation of multiple loci. P-OB performed the heterogeneity analyses. HG and GJo carried out the array-CGH analyses. OThJ, BAA, RBB, AA, HN, RW, ÅB, PM, BM, KP, AM, TH, KA and CB provided samples and information on the families included in this study. AA, with the help of RBB, wrote the manuscript and all co-authors critically read and approved it. RBB conceived and coordinated the study.

## Supplementary Material

Additional file 1 **Figure S1, pedigrees of the families in the GWS**. This is a jpg file showing pedigrees of the families included in the GWS. Pedigrees of nine Icelandic non-*BRCA1/2 *families with each showing BC cases traced to a single pair of founders but otherwise omitting relatives if not genotyped. Genotyped family members are marked with an asterisk. Circles denote females and boxes males, with red filling denoting diagnosis of BC and shaded red also ovarian cancer. Tan filling indicates cancer at other sites than breast, or of unknown origin. Information about the site and approximate age (in years) at diagnosis of cancer is shown below the symbols (Br for breast, Col colon, Cvx cervix, Kdn kidney, Lng lung, Ov ovary, Pnc pancreas, Pro prostate, Stm stomach and Unkn for unknown origin). Dotted vertical lines between family branches show how the family was separated in two parts for linkage calculations. Pedigrees are somewhat distorted in order to avoid recognition. Click here for file

Additional file 2 **Supplementary methods**. A Word document containing a methodological description of intra-family significance testing for multiple loci. Click here for file

Additional file 3 **Figure S2, parametric LOD scores by family**. A jpg file showing graphs of parametric LOD scores by chromosomal position, for the families in the GWS (the top graph with all families combined, for comparison). LOD scores are shown for the dominant (dark teal line) and the recessive model (plum). Three families (70070, 70228 and 70236) were separated in smaller units for linkage analysis, as indicated by adding the letter a or b to the family name. Click here for file

Additional file 4 **Figure S3, NP-LOD scores by family**. A jpg file showing graphs of NP-LOD scores and associated *P*-values by chromosomal position in individual families included in GWS, using different exponential scoring options in Merlin software: S-all (orange thick line) and S-pairs (indigo). Three families (70070, 70228 and 70236) were separated in smaller units for linkage analysis, as indicated by adding the letter a or b to the family name. Click here for file

Additional file 5 **Table S1, Maximum LODs by chromosome and family, for NP-LODs with *P *< 0.005**. A Word file containing a table of per-family LOD signals (selected with respect to NP-LOD associated *P*-values), for consideration of whether any chromosomal positions may be indicated by more than one family. Click here for file

## References

[B1] Stratton MR Rahman N The emerging landscape of breast cancer susceptibility Nat Genet 2008 40 17 22 10.1038/ng.2007.53 18163131

[B2] Anglian Breast Cancer Study Group Prevalence and penetrance of BRCA1 and BRCA2 mutations in a population-based series of breast cancer cases. Anglian Breast Cancer Study Group Br J Cancer 2000 83 1301 1308 11044354 10.1054/bjoc.2000.1407 PMC2408797

[B3] Arason A Jonasdottir A Barkardottir RB Bergthorsson JT Teare MD Easton DF Egilsson V A population study of mutations and LOH at breast cancer gene loci in tumours from sister pairs: two recurrent mutations seem to account for all BRCA1/BRCA2 linked breast cancer in Iceland J Med Genet 1998 35 446 449 9643283 10.1136/jmg.35.6.446 PMC1051336

[B4] Ford D Easton DF Stratton M Narod S Goldgar D Devilee P Bishop DT Weber B Lenoir G Chang-Claude J Sobol H Teare MD Struewing J Arason A Scherneck S Peto J Rebbeck TR Tonin P Neuhausen S Barkardottir R Eyfjord J Lynch H Ponder BA Gayther SA Zelada-Hedman M The Breast Cancer Linkage Consortium Genetic heterogeneity and penetrance analysis of the BRCA1 and BRCA2 genes in breast cancer families Am J Hum Genet 1998 62 676 689 9497246 10.1086/301749 PMC1376944

[B5] Peto J Collins N Barfoot R Seal S Warren W Rahman N Easton DF Evans C Deacon J Stratton MR Prevalence of BRCA1 and BRCA2 gene mutations in patients with early-onset breast cancer J Natl Cancer Inst 1999 91 943 949 10.1093/jnci/91.11.943 10359546

[B6] Antoniou AC Pharoah PP Smith P Easton DF The BOADICEA model of genetic susceptibility to breast and ovarian cancer Br J Cancer 2004 91 1580 1590 15381934 10.1038/sj.bjc.6602175 PMC2409934

[B7] Cui J Antoniou AC Dite GS Southey MC Venter DJ Easton DF Giles GG McCredie MR Hopper JL After BRCA1 and BRCA2-what next? Multifactorial segregation analyses of three-generation, population-based Australian families affected by female breast cancer Am J Hum Genet 2001 68 420 431 11133358 10.1086/318187 PMC1235275

[B8] Ponder BA Antoniou A Dunning A Easton DF Pharoah PD Polygenic inherited predisposition to breast cancer Cold Spring Harb Symp Quant Biol 2005 70 35 41 10.1101/sqb.2005.70.029 16869736

[B9] Meindl A Hellebrand H Wiek C Erven V Wappenschmidt B Niederacher D Freund M Lichtner P Hartmann L Schaal H Ramser J Honisch E Kubisch C Wichmann HE Kast K Deissler H Engel C Muller-Myhsok B Neveling K Kiechle M Mathew CG Schindler D Schmutzler RK Hanenberg H Germline mutations in breast and ovarian cancer pedigrees establish RAD51C as a human cancer susceptibility gene Nat Genet 42 410 414 10.1038/ng.569 20400964

[B10] Huusko P Juo SH Gillanders E Sarantaus L Kainu T Vahteristo P Allinen M Jones M Rapakko K Eerola H Markey C Vehmanen P Gildea D Freas-Lutz D Blomqvist C Leisti J Blanco G Puistola U Trent J Bailey-Wilson J Winqvist R Nevanlinna H Kallioniemi OP Genome-wide scanning for linkage in Finnish breast cancer families Eur J Hum Genet 2004 12 98 104 10.1038/sj.ejhg.5201091 14560309

[B11] Smith P McGuffog L Easton DF Mann GJ Pupo GM Newman B Chenevix-Trench G Szabo C Southey M Renard H Odefrey F Lynch H Stoppa-Lyonnet D Couch F Hopper JL Giles GG McCredie MR Buys S Andrulis I Senie R Goldgar DE Oldenburg R Kroeze-Jansema K Kraan J Meijers-Heijboer H Klijn JG van Asperen C van Leeuwen I Vasen HF Cornelisse CJ A genome wide linkage search for breast cancer susceptibility genes Genes Chromosomes Cancer 2006 45 646 655 16575876 10.1002/gcc.20354 PMC2714969

[B12] Bergman A Karlsson P Berggren J Martinsson T Bjorck K Nilsson S Wahlstrom J Wallgren A Nordling M Genome-wide linkage scan for breast cancer susceptibility loci in Swedish hereditary non-BRCA1/2 families: suggestive linkage to 10q23.32-q25.3 Genes Chromosomes Cancer 2007 46 302 309 10.1002/gcc.20405 17171685

[B13] Gonzalez-Neira A Rosa-Rosa JM Osorio A Gonzalez E Southey M Sinilnikova O Lynch H Oldenburg RA van Asperen CJ Hoogerbrugge N Pita G Devilee P Goldgar D Benitez J Genomewide high-density SNP linkage analysis of non-BRCA1/2 breast cancer families identifies various candidate regions and has greater power than microsatellite studies BMC Genomics 2007 8 299 17760956 10.1186/1471-2164-8-299 PMC2072960

[B14] Oldenburg RA Kroeze-Jansema KH Houwing-Duistermaat JJ Bayley JP Dambrot C van Asperen CJ van den Ouweland AM Bakker B van Beers EH Nederlof PM Vasen H Hoogerbrugge N Cornelisse CJ Meijers-Heijboer H Devilee P Genome-wide linkage scan in Dutch hereditary non-BRCA1/2 breast cancer families identifies 9q21-22 as a putative breast cancer susceptibility locus Genes Chromosomes Cancer 2008 47 947 956 10.1002/gcc.20597 18663745

[B15] Rosa-Rosa JM Pita G Gonzalez-Neira A Milne RL Fernandez V Ruivenkamp C van Asperen CJ Devilee P Benitez J A 7 Mb region within 11q13 may contain a high penetrance gene for breast cancer Breast Cancer Res Treat 2009 118 151 159 10.1007/s10549-009-0317-1 19205878

[B16] Rosa-Rosa JM Pita G Urioste M Llort G Brunet J Lazaro C Blanco I Ramon y Cajal T Diez O de la Hoya M Caldes T Tejada MI Gonzalez-Neira A Benitez J Genome-wide linkage scan reveals three putative breast-cancer-susceptibility loci Am J Hum Genet 2009 84 115 122 19147119 10.1016/j.ajhg.2008.12.013 PMC2668009

[B17] Kainu T Juo SH Desper R Schaffer AA Gillanders E Rozenblum E Freas-Lutz D Weaver D Stephan D Bailey-Wilson J Kallioniemi OP Tirkkonen M Syrjakoski K Kuukasjarvi T Koivisto P Karhu R Holli K Arason A Johannesdottir G Bergthorsson JT Johannsdottir H Egilsson V Barkardottir RB Johannsson O Haraldsson K Sandberg T Holmberg E Gronberg H Olsson H Borg A Somatic deletions in hereditary breast cancers implicate 13q21 as a putative novel breast cancer susceptibility locus Proc Natl Acad Sci USA 2000 97 9603 9608 10944226 10.1073/pnas.97.17.9603 PMC16911

[B18] Seitz S Rohde K Bender E Nothnagel A Kolble K Schlag PM Scherneck S Strong indication for a breast cancer susceptibility gene on chromosome 8p12-p22: linkage analysis in German breast cancer families Oncogene 1997 14 741 743 10.1038/sj.onc.1200881 9038382

[B19] Zuppan P Hall JM Lee MK Ponglikitmongkol M King MC Possible linkage of the estrogen receptor gene to breast cancer in a family with late-onset disease Am J Hum Genet 1991 48 1065 1068 2035527 PMC1683085

[B20] Smith SA Easton DF Evans DG Ponder BA Allele losses in the region 17q12-21 in familial breast and ovarian cancer involve the wild-type chromosome Nat Genet 1992 2 128 131 10.1038/ng1092-128 1303261

[B21] Gudmundsson J Johannesdottir G Bergthorsson JT Arason A Ingvarsson S Egilsson V Barkardottir RB Different tumor types from BRCA2 carriers show wild-type chromosome deletions on 13q12-q13 Cancer Res 1995 55 4830 4832 7585515

[B22] Knudson AG Jr Mutation and cancer: statistical study of retinoblastoma Proc Natl Acad Sci USA 1971 68 820 823 5279523 10.1073/pnas.68.4.820 PMC389051

[B23] Bergthorsson JT Jonasdottir A Johannesdottir G Arason A Egilsson V Gayther S Borg A Hakanson S Ingvarsson S Barkardottir RB Identification of a novel splice-site mutation of the BRCA1 gene in two breast cancer families: screening reveals low frequency in Icelandic breast cancer patients Hum Mutat 1998 Suppl 1 S195 197 9452084 10.1002/humu.1380110163

[B24] Johannesdottir G Gudmundsson J Bergthorsson JT Arason A Agnarsson BA Eiriksdottir G Johannsson OT Borg A Ingvarsson S Easton DF Egilsson V Barkardottir RB High prevalence of the 999del5 mutation in icelandic breast and ovarian cancer patients Cancer Res 1996 56 3663 3665 8706004

[B25] Thorlacius S Sigurdsson S Bjarnadottir H Olafsdottir G Jonasson JG Tryggvadottir L Tulinius H Eyfjord JE Study of a single BRCA2 mutation with high carrier frequency in a small population Am J Hum Genet 1997 60 1079 1084 9150155 PMC1712443

[B26] Miller SA Dykes DD Polesky HF A simple salting out procedure for extracting DNA from human nucleated cells Nucleic Acids Res 1988 16 1215 3344216 10.1093/nar/16.3.1215 PMC334765

[B27] Jonsson G Staaf J Olsson E Heidenblad M Vallon-Christersson J Osoegawa K de Jong P Oredsson S Ringner M Hoglund M Borg A High-resolution genomic profiles of breast cancer cell lines assessed by tiling BAC array comparative genomic hybridization Genes Chromosomes Cancer 2007 46 543 558 10.1002/gcc.20438 17334996

[B28] Abecasis GR Cherny SS Cookson WO Cardon LR Merlin--rapid analysis of dense genetic maps using sparse gene flow trees Nat Genet 2002 30 97 101 10.1038/ng786 11731797

[B29] Easton DF Bishop DT Ford D Crockford GP the Breast Cancer Linkage Consortium Genetic linkage analysis in familial breast and ovarian cancer: results from 214 families Am J Hum Genet 1993 52 678 701 8460634 PMC1682082

[B30] Claus EB Risch N Thompson WD Genetic analysis of breast cancer in the cancer and steroid hormone study Am J Hum Genet 1991 48 232 242 1990835 PMC1683001

[B31] Whittemore AS Halpern J A class of tests for linkage using affected pedigree members Biometrics 1994 50 118 127 10.2307/2533202 8086596

[B32] Weeks DE Lange K The affected-pedigree-member method of linkage analysis Am J Hum Genet 1988 42 315 326 3422543 PMC1715269

[B33] Kong A Cox NJ Allele-sharing models: LOD scores and accurate linkage tests Am J Hum Genet 1997 61 1179 1188 9345087 10.1086/301592 PMC1716027

[B34] Matise TC Chen F Chen W De La Vega FM Hansen M He C Hyland FC Kennedy GC Kong X Murray SS Ziegle JS Stewart WC Buyske S A second-generation combined linkage physical map of the human genome Genome Res 2007 17 1783 1786 17989245 10.1101/gr.7156307 PMC2099587

[B35] Kong A Gudbjartsson DF Sainz J Jonsdottir GM Gudjonsson SA Richardsson B Sigurdardottir S Barnard J Hallbeck B Masson G Shlien A Palsson ST Frigge ML Thorgeirsson TE Gulcher JR Stefansson K A high-resolution recombination map of the human genome Nat Genet 2002 31 241 247 12053178 10.1038/ng917

[B36] Lander E Kruglyak L Genetic dissection of complex traits: guidelines for interpreting and reporting linkage results Nat Genet 1995 11 241 247 10.1038/ng1195-241 7581446

