# Supplementary figures and images for: Genome-wide search for breast cancer linkage in large Icelandic non-BRCA1/2 families
Source: Breast Cancer Res. 2010 Jul 16;12(4):R50. doi: 10.1186/bcr2608 (PMC2949638; doi:10.1186/bcr2608)

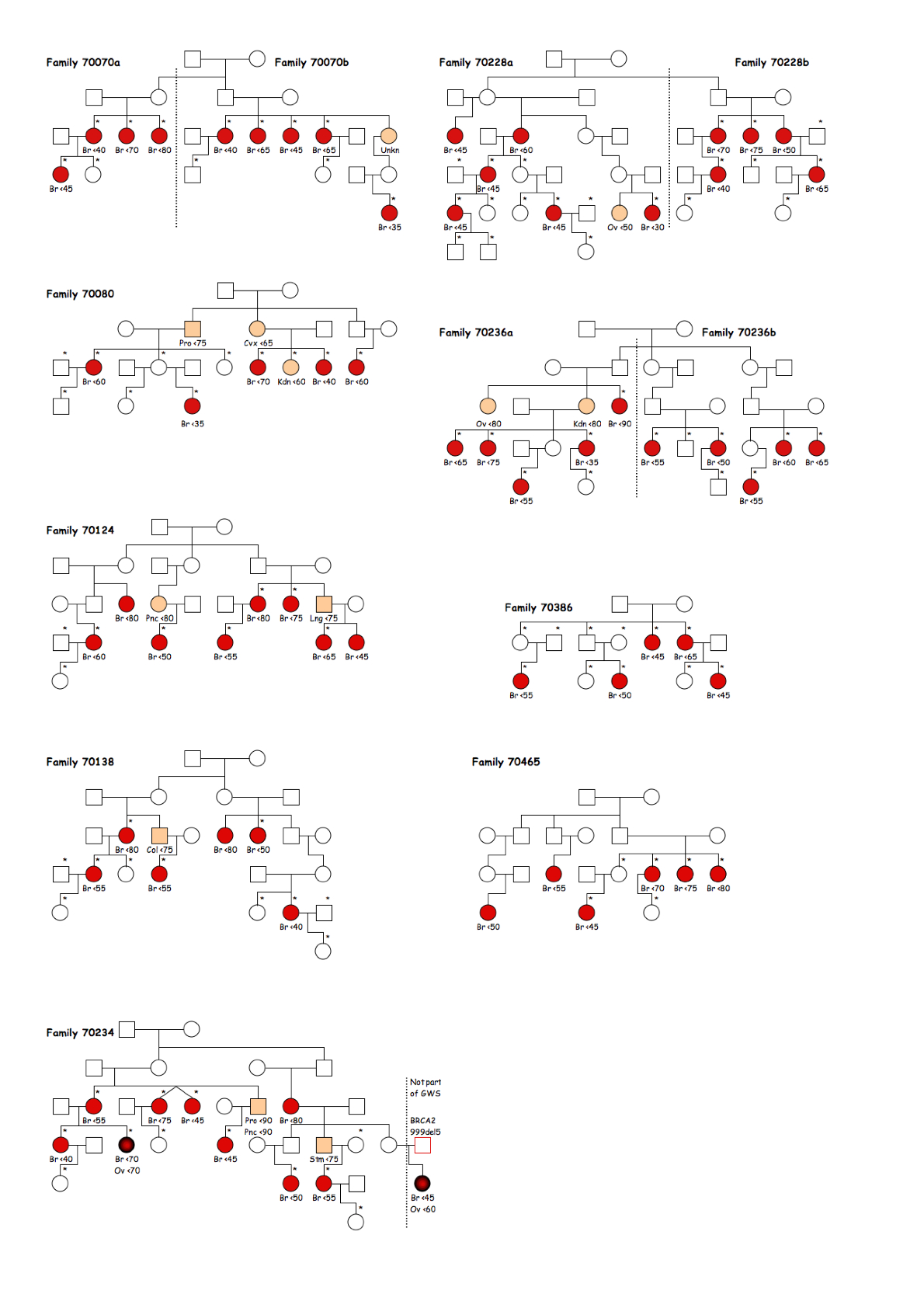

Supplement: Additional file 1 — Figure S1, pedigrees of the families in the GWS. This is a jpg file showing pedigrees of the families included in the GWS. Pedigrees of nine Icelandic non-BRCA1/2 families with each showing BC cases traced to a single pair of founders but otherwise omitting relatives if not genotyped. Genotyped family members are marked with an asterisk. Circles denote females and boxes males, with red filling denoting diagnosis of BC and shaded red also ovarian cancer. Tan filling indicates cancer at other sites than breast, or of unknown origin. Information about the site and approximate age (in years) at diagnosis of cancer is shown below the symbols (Br for breast, Col colon, Cvx cervix, Kdn kidney, Lng lung, Ov ovary, Pnc pancreas, Pro prostate, Stm stomach and Unkn for unknown origin). Dotted vertical lines between family branches show how the family was separated in two parts for linkage calculations. Pedigrees are somewhat distorted in order to avoid recognition. [file bcr2608-S1.jpeg]

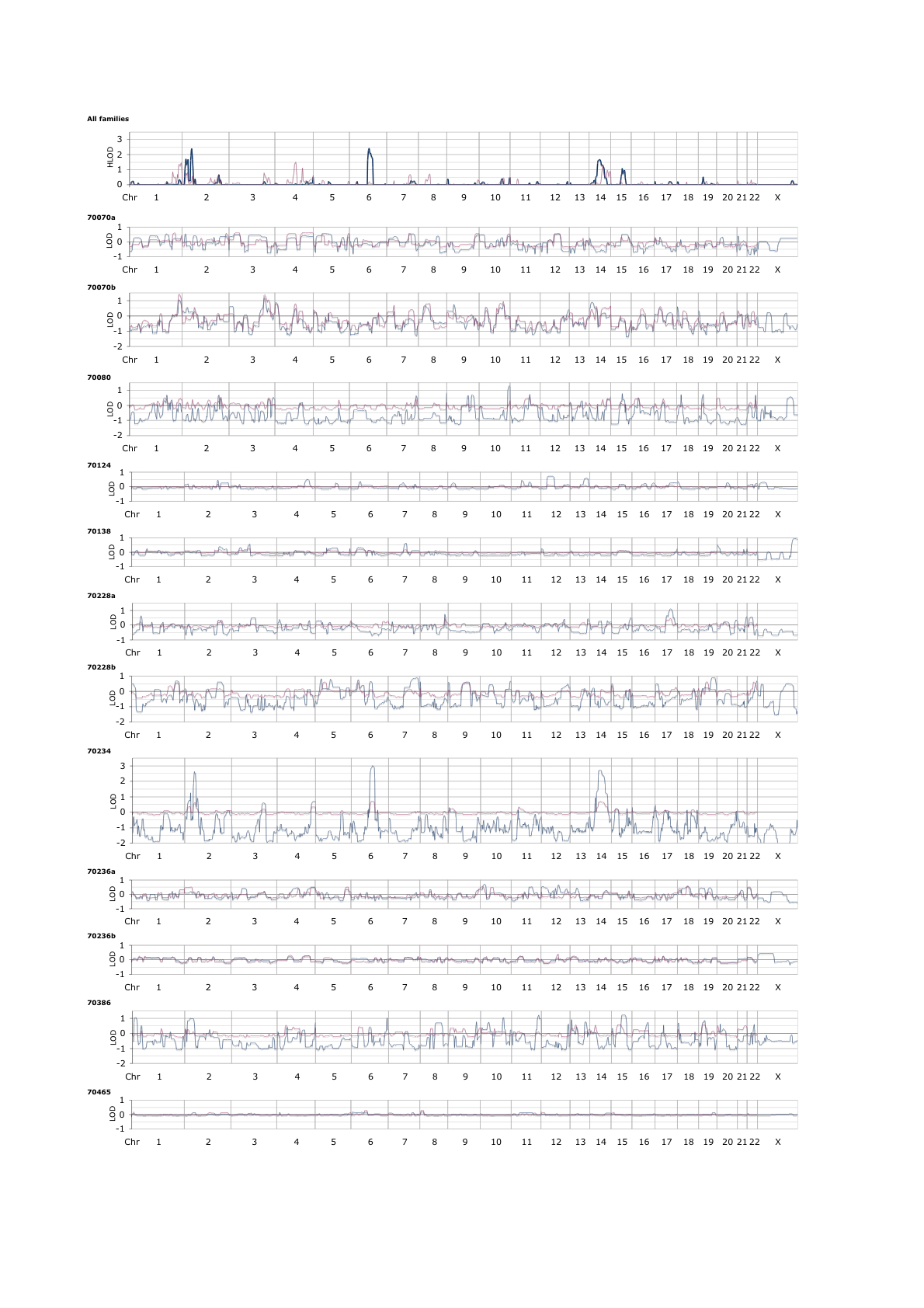

Supplement: Additional file 3 — Figure S2, parametric LOD scores by family. A jpg file showing graphs of parametric LOD scores by chromosomal position, for the families in the GWS (the top graph with all families combined, for comparison). LOD scores are shown for the dominant (dark teal line) and the recessive model (plum). Three families (70070, 70228 and 70236) were separated in smaller units for linkage analysis, as indicated by adding the letter a or b to the family name. [file bcr2608-S3.jpeg]

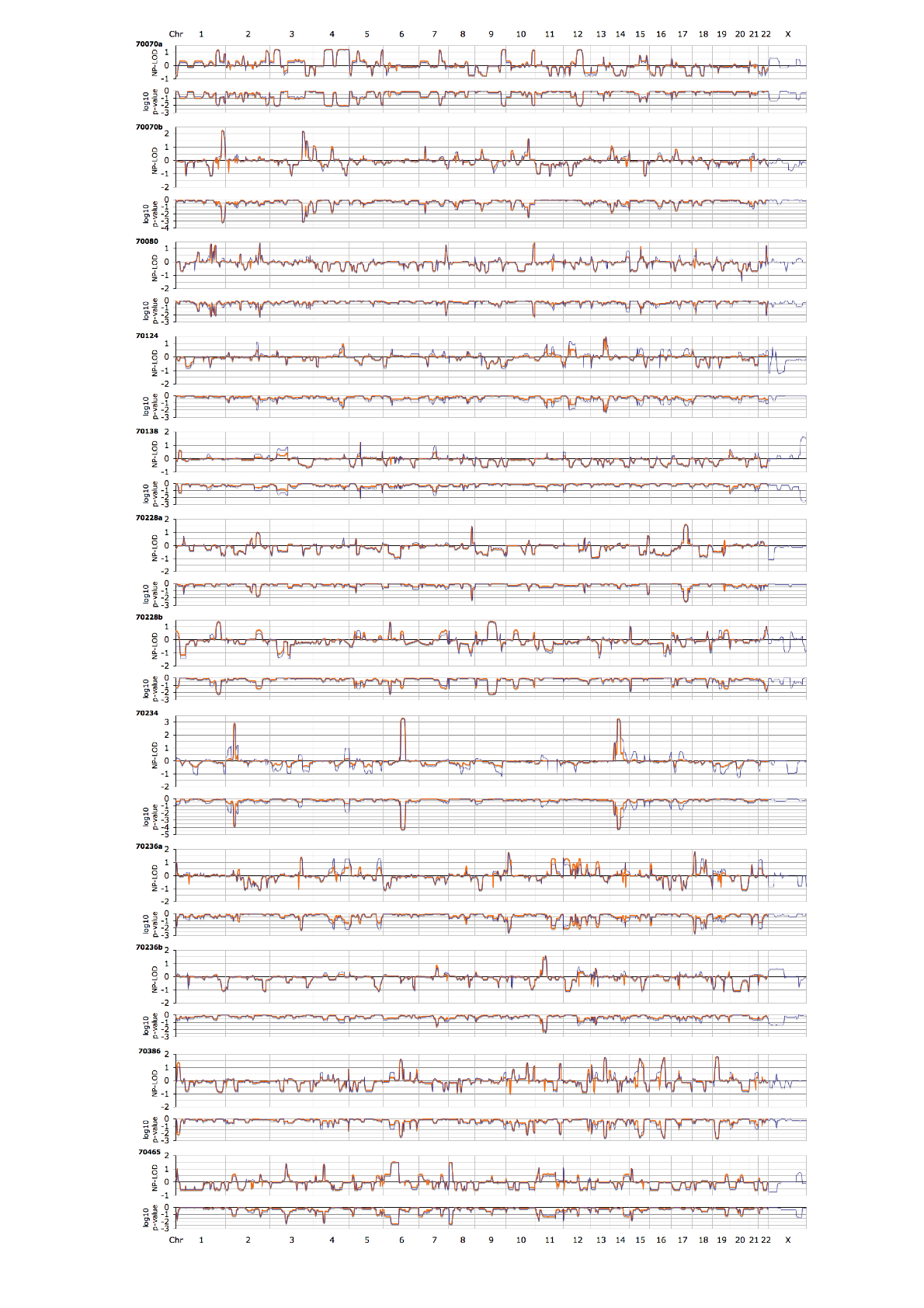

Supplement: Additional file 4 — Figure S3, NP-LOD scores by family. A jpg file showing graphs of NP-LOD scores and associated P-values by chromosomal position in individual families included in GWS, using different exponential scoring options in Merlin software: S-all (orange thick line) and S-pairs (indigo). Three families (70070, 70228 and 70236) were separated in smaller units for linkage analysis, as indicated by adding the letter a or b to the family name. [file bcr2608-S4.jpeg]
