# Supplementary material for: Genome-wide search for breast cancer linkage in large Icelandic non-BRCA1/2 families
Source: Breast Cancer Res. 2010 Jul 16;12(4):R50. doi: 10.1186/bcr2608 (PMC2949638; doi:10.1186/bcr2608)
Supplement: Additional file 2 — Supplementary methods. A Word document containing a methodological description of intra-family significance testing for multiple loci. [file bcr2608-S2.doc]

*Intra-family significance testing for multiple loci*

For a multi-generation pedigree with a single pair of ungenotyped founders, the probability of a fixed locus being identical by descent in all affected individuals through *m* meioses is 4 × 0.5*^m^* = 0.5*^m^*^-2^ (the multiplication by 4 is because each outcome is a 50:50 test, but two of them are sure to be successes, (i) the first considered meiosis, determining which allele/haplotype to test for, and (ii) the first comparison to the founder pair, since it involves all four possible chromosomal origins). For cosegregation of two positionally independent loci (assuming no interference), the probability is squared and becomes 0.25*^m^*^-2^ and for three loci it is 0.125*^m^*^-2^.

As a step towards allowing for *a priori* unknown positions, not linked to each other, an estimate of the number of unlinked intervals in the human genome could be obtained by dividing the total map length (all chromosomes combined) by 50 cM, because for loci further apart there is a 50% chance of recombination. Depending on whether male or female map length is used, one obtains 58 or 92 intervals, but in order to simplify and be a little conservative, we choose to use the number 100.

If *i* is the total number of independent genomic positions derived as above, the number of possible pairings of any two is the binomial coefficient *i*(*i*-1)/2. The probability 0.25*^m^*^-2^ should be multiplied by this, giving in our case 4950 × 0.25*^m^*^-2^. Strictly, correction should also be made allowing for the probability of all less likely combinations (not only pairs but 3, 4, etc. jointly segregated loci), but this is negligible. For cosegregation of three loci the corresponding multiplier is *i*(*i*-1)(*i*-2)/6.

Finally, if one family indicates multiple genes, a Bonferroni adjustment should be applied to the probability estimate, because several families were included in the GWS. When doing this, one should allow for the different sizes of the families. A fair correction would be to multiply with the number of families with similar or greater total number of meioses. If N is the number of such families, the resulting p-value for two loci (and *i* = 100) is

p = N × 4950 × 0.25*^m^*^-2^ (1)

and for three loci

p = N × 161700 × 0.125*^m^*^-2^ (2)
