# Supplementary material for: Genome-wide search for breast cancer linkage in large Icelandic non-BRCA1/2 families
Source: Breast Cancer Res. 2010 Jul 16;12(4):R50. doi: 10.1186/bcr2608 (PMC2949638; doi:10.1186/bcr2608)
Supplement: Additional file 5 — Table S1, Maximum LODs by chromosome and family, for NP-LODs with P < 0.005. A Word file containing a table of per-family LOD signals (selected with respect to NP-LOD associated P-values), for consideration of whether any chromosomal positions may be indicated by more than one family. [file bcr2608-S5.doc]

# Supplementary Table S1

# Maximum LOD scores (dominant model) by chromosome and family, for NP-LODs with p<0.005

| Chromosome | Region | Family | LOD | NP-LOD (p-value) | Marker |
| --- | --- | --- | --- | --- | --- |
| 1 | q43 | 70070b | 1.04 | 2.26 (0.0006) | D1S304 |
| 2 | p22.3 | 70234 | 2.63 | 2.94 (0.00012) | D2S367 |
| 3 | q26.2 | 70070b | 1.15 | 2.20 (0.0007) | D3S1614 |
|  | q27.1 | 70070b | 0.50 | 1.47 (0.0047) | D3S3609 |
| 6 | p21.1 | 70465 | 0.14 | 1.50 (0.004) | D6S282 |
|  | q15 (q14.2-q16.1) | 70386 | 0.44 | 1.61 (0.003) | D6S462 |
|  | q16.3 (q15-q22.31) | 70234 | 3.03 | 3.31 (0.00005) | D6S434 |
| 8 | p23.1 | 70465 | 0.17 | 1.49 (0.004) | D8S503 |
|  | q24.22 | 70228a | 0.74 | 1.47 (0.0047) | D8S256 |
| 10 | p14 | 70236a | 0.66 | 1.78 (0.002) | D10S189 |
|  | q25.1 | 70070b | 0.76 | 1.64 (0.003) | D10S597 |
|  | q26.3 | 70080 | 1.23 | 1.45 (0.0049) | D10S212 |
| 11 | p12 | 70236b | 0.22 | 1.58 (0.004) | D11S4102 |
| 13 | q32.2 (q32.1-q33.1) | 70386 | 0.77 | 1.74 (0.002) | D13S159 |
|  | q33.1(q31.3-q33.1) | 70124 | 0.61 | 1.45 (0.0048) | D13S158 |
| 14 | q23.2 | 70234 | 2.74 | 3.25 (0.00005) | D14S63 |
| 15 | q22.1 | 70386 | 1.23 | 1.69 (0.003) | D15S117 |
| 16 | q21 | 70386 | 0.73 | 1.74 (0.002) | D16S514 |
| 17 | q24.2 | 70228a | 1.11 | 1.64 (0.003) | D17S1816 |
| 18 | p11.31 | 70236a | 0.31 | 1.86 (0.002) | D18S1132 |
| 19 | p13.2-p13.13 | 70386 | 0.87 | 1.78 (0.002) | D19S221 |
| X | q27.3-qter | 70138 | 0.94 | 1.62 (0.003) | DXS8045 |
